# A Polygonatum-Based Functional Formula Improves Stress-Induced Depressive-like Behaviors via Modulation of Neuroinflammation and Tryptophan Metabolism

**DOI:** 10.3390/foods15060973

**Published:** 2026-03-10

**Authors:** Guyue Zhou, Ning Jiang, Jixian Liu, Xiangjunlin Zhang, Yanfei Xu, Xinmin Liu, Mengzhou Xie

**Affiliations:** 1 School of Traditional Chinese Medicine, Hunan University of Chinese Medicine, Changsha 410208, China; 13451239980@163.com; 2 Sino-Pakistan Center on Traditional Chinese Medicine, Hunan University of Medicine, Huaihua 418000, China; jiangning0603@163.com; 3 Research Center for Pharmacology and Toxicology, Institute of Medicinal Plant Development (IMPLAD), Chinese Academy of Medical Sciences and Peking Union Medical College, Beijing 100193, China; 18784021235@163.com (X.Z.); 18385492847@163.com (Y.X.); 4 Institute of Drug Discovery Technology, Ningbo University, Ningbo 315211, China; liujixian_caresse@163.com

**Keywords:** functional food, depression, neuroinflammation, tryptophan metabolism, pyroptosis

## Abstract

Depression-related mood disturbances are increasingly recognized as nutrition-sensitive conditions associated with chronic stress-induced neuroinflammation and metabolic imbalance. *Polygonatum sibiricum*, *Poria cocos*, *Lilium brownii*, and *Radix Glycyrrhizae Preparata* are edible medicinal plants commonly used in functional foods. In this study, we evaluated the antidepressant effects of a *Polygonatum sibiricum*-based functional formula (PSF) in a chronic restraint stress (CRS) mouse model. CRS induced prominent anhedonia and behavioral despair, accompanied by microglial overactivation, activation of the NLRP3 inflammasome, and dysregulated tryptophan metabolism. PSF supplementation significantly alleviated depressive-like behaviors and inhibited NLRP3–caspase-1–GSDMD-mediated pyroptosis, leading to reduced hippocampal IL-1β and IL-18 levels. Importantly, PSF restored tryptophan metabolism toward serotonin production, stabilized monoaminergic and glutamate/GABA neurotransmission, and protected hippocampal neurons. Moreover, PSF partially reversed stress-induced gut microbiota dysbiosis. Collectively, these results demonstrate that PSF acts as a neuroimmune–metabolic modulator that improves mood-related behaviors by regulating inflammatory signaling, tryptophan metabolism, and neurotransmitter homeostasis, supporting its potential development as a functional food intervention for stress-induced depression.

## 1. Introduction

Depression is characterized by elevated morbidity, significant disability, and increased mortality rates, resulting in profound harm to patients and imposing substantial burdens on families and society, thus emerging as a critical global public health issue and a prominent social concern [1]. Depression has become one of the most prevalent mental diseases, representing about one-third of the social and economic burdens associated with worldwide disease. It is anticipated to become the most socioeconomically burdensome disease globally by 2030 [2]. The primary treatment for depression involves oral antidepressants, notably serotonin reuptake inhibitors (SSRIs) and serotonin–norepinephrine reuptake inhibitors (SNRIs). While these medications can alleviate symptoms to a certain extent, prolonged use frequently results in side effects, including adverse reactions and withdrawal symptoms. Consequently, there is an immediate need to investigate safer and sustainable alternatives for intervention [3]. Recent evidence increasingly suggests that neuroinflammation and metabolic dysregulation play important roles in stress-related depressive-like behaviors. Among these processes, NLRP3 inflammasome–associated inflammatory signaling and alterations in tryptophan–kynurenine metabolism are considered closely interconnected pathways [4,5]. Activation of the NLRP3 inflammasome complex, comprising NLRP3, apoptosis-associated speck-like protein (ASC), and caspase-1, can trigger gasdermin-mediated pyroptosis and promote the maturation and release of pro-inflammatory cytokines such as interleukin-1β (IL-1β) and interleukin-18 (IL-18), thereby amplifying neuroinflammatory responses [6,7,8,9,10,11]. In parallel, inflammatory activation may reshape cerebral metabolic homeostasis, particularly by shifting tryptophan metabolism from the serotonin (5-HT) synthesis pathway toward the kynurenine pathway through the induction of indoleamine-2,3-dioxygenase (IDO). This metabolic shift has been associated with reduced central 5-HT availability and increased production of neuroactive kynurenine metabolites, which are implicated in stress-induced behavioral alterations [12]. In this context, dietary interventions and functional foods have attracted increasing attention as safe and sustainable approaches for supporting neuroimmune and metabolic balance [13]. Edible medicinal plants are rich sources of bioactive compounds capable of modulating inflammatory signaling, neurotransmitter systems, and metabolic pathways [14,15,16]. Compared with single-target pharmacological agents, multi-component functional formulations may exert synergistic regulatory effects across immune, metabolic, and neural systems, highlighting their potential value in the dietary management of stress-related mood disturbances.

Functional foods derived from edible plants exert health benefits through the synergistic actions of multiple bioactive components, enabling coordinated regulation of complex physiological processes. Such multi-component characteristics provide advantages in modulating chronic, multifactorial conditions, including stress-related mood disturbances, by influencing inflammatory responses, neurotransmitter metabolism, and energy homeostasis [17,18,19]. *Polygonatum sibiricum*, known as Huangjing in Chinese, is a traditional medicinal and edible plant that has been used for more than 2000 years in East Asian medicine. In classical materia medica, including *Shennong’s Herbal Classic* and *Ben Cao Gang Mu*, Huangjing is classified as a superior-grade herb and traditionally prescribed to nourish yin, tonify qi, replenish essence, and calm the spirit. According to Traditional Chinese Medicine (TCM) theory, emotional disorders such as melancholic syndrome and “lily bulb disease” are commonly associated with a deficiency of qi and yin, mental fatigue, and impaired nourishment of the mind [20,21]. Huangjing-containing prescriptions have long been used to alleviate fatigue, emotional instability, and stress-related symptoms, indicating their traditional relevance to emotional and mental disorders [21]. The *Polygonatum sibiricum*-based functional formula (PSF) is a core clinical empirical prescription developed by Dr. Pei Hui from Xiyuan Hospital, China Academy of Chinese Medical Sciences. PSF used in this study consists of *Polygonatum sibiricum*, *Poria cocos*, *Lilium brownii,* and *Radix Glycyrrhizae Preparata* in a ratio of 3:5:5:1. These four food–medicinal homologous plants have been consumed for centuries in East Asia and are commonly incorporated into dietary preparations aimed at supporting emotional balance and vitality. Modern studies indicate that their major bioactive constituents, including polysaccharides, saponins, and flavonoids, possess anti-inflammatory, neuromodulatory, and metabolic regulatory properties, with reported effects on stress-related behavioral abnormalities, monoaminergic neurotransmission, and neurotrophic signaling pathways [22,23,24,25]. Collectively, these findings suggest that PSF may exert mood-regulating effects through the coordinated dietary modulation of neuroimmune and metabolic pathways. However, whether a plant-based formula derived from these materials can coordinately modulate tryptophan–kynurenine metabolism and NLRP3-associated neuroinflammation under chronic stress conditions remains unclear. Therefore, this study employed a chronic restraint stress (CRS) mouse model to investigate the nutrition-driven mechanisms of PSF in regulating stress-related depressive-like behaviors and to evaluate its potential as a dietary functional food strategy for supporting emotional health.

## 2. Materials and Methods

### 2.1. Animals

A total of 96 male ICR mice (weighing 28 ± 2 g), SPF grade, were obtained from Charles River Laboratories in Beijing, China, under the qualification number SCXK(Jing) 2023-0049. During the experiment, the animals were housed in SPF-rated facilities, had unrestricted access to standard feed and potable water, experienced a 12 h light/dark cycle, and were maintained at a temperature of 25 ± 2 °C and a humidity level of 55 ± 10%. The model was established after 7 days of routine feeding. This experimental protocol has been reviewed and approved by the Laboratory Animal Management and Animal Welfare Committee (IACUC) of the Institute of Medicinal Plants, Chinese Academy of Medical Sciences (SLXD-20250424022).

### 2.2. Experimental Reagents

*Polygonatum sibiricum* (2023-122-683), *Poria cocos* (2024-122-693), *Lilium brownii* (2024-122-505), and *Radix Glycyrrhizae Preparata* (2023-122-438) (identified by Professor Zhiying Yuan, College of Pharmacy, Hunan University of Chinese Medicine). Fluoxetine hydrochloride (F131623, Aladdin Reagents Ltd., Shanghai, China); sucrose (Beijing chemical plant, Beijing, China); Whole protein extraction kit (Beijing Solaibao Technology Co., Ltd., Beijing, China, BC3710); BCA Protein Assay Kit (Jiangsu Kangwei Century Biotechnology Co., Ltd., Taizhou, China, CW0014S); SDS-PAGE Gel Preparation Kit (Jiangsu Kangwei Century Biotechnology Co., Ltd., Taizhou, China, CW0027S); Western Blot Membrane Regeneration Liquid (Jiangsu Kangwei Century Biotechnology Co., Ltd., Taizhou, China, CW0056M); GSDMD Antibody (CST Corporation, Houston, TX, USA, 39754S); Caspase1 antibody (CST, Houston, TX, USA, 24232S); NLRP3 antibody (CST Corporation, Houston, TX, USA, 15101S); IL-18 (CST Corporation, Houston, TX, USA, 87058S); ASC antibody (Invitrogen, Carlsbad, CA, USA, PA5-95826); IL-1β antibody (Wuhan Mitaka Biotechnology Co., Ltd., Wuhan, China, 26048-1-AP); β-actin antibody (Wuhan Mitaka Biotechnology Co., Ltd., Wuhan, China,200536-1-AP); HRP Goat Anti-Rabbit IgG (Wuhan Aibtech Biotechnology Co., Ltd., Wuhan, China, 67314). IgGTRIzon total RNA extraction reagent (Jiangsu Kangwei Century Biotechnology Co., Ltd., Taizhou, China, CW0580S). TransScript^®^ II All-in-One First-Strand cDNA Synthesis SuperMix for qPCR (One-Step gDNA Removal, Beijing, China) AT341-02), PerfectStart Green qPCR SuperMix (AQ601-02-V2), Beijing Quanshijin Biotechnology (TransGen Biotech) Co., Ltd., Beijing, China, Chromatographic grade formic acid (batch number: 2023041701) and analytical pure methanol (batch number: 2022092002) were purchased from Chengdu Cologne Chemical Co., Ltd., Chengdu, China; Chromatographic grade acetonitrile (Thermo Fisher Scientific Co., Ltd., Waltham, MA, USA, batch number: F22M8G206; Distilled Water (provided by Watsons Food & Beverage Co., Ltd., Hong Kong, China, 20240516). Primers (provided by Generay, Shanghai, China). The gene sequences are shown in the Table 1 below.

### 2.3. Experimental Methods

#### 2.3.1. Sample Preparation and Analysis

**Figure 1:**
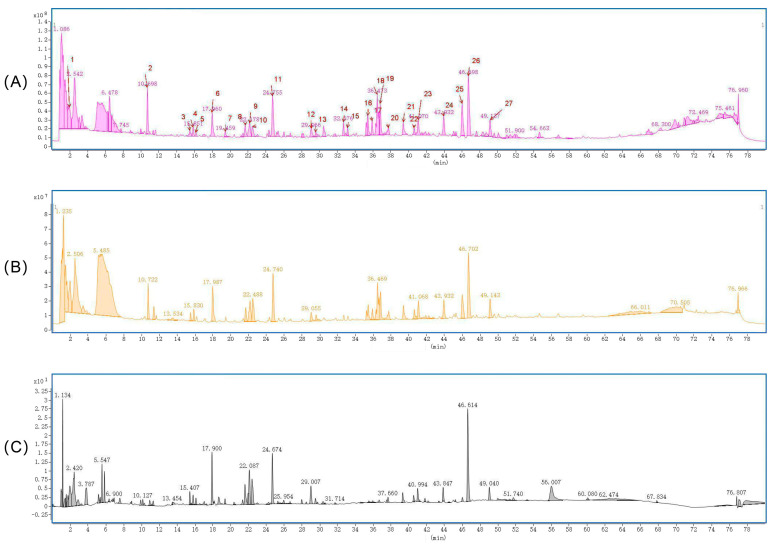
LCMS characterization of PSF components. (**A**) Total ion current chromatogram of PSF in positive mode; (**B**) total ion current chromatography of PSF in negative mode; (**C**) UV absorption diagram of PSF at 254 mm wavelength.

*Polygonatum sibiricum*, *Poria cocos*, *Lilium brownii,* and processed *Radix Glycyrrhizae Preparata* were used to perform three cycles of water extraction using distilled water in a ratio of 3:5:5:1. The solution was filtered through a 100-mesh sieve and then concentrated to a final concentration of 1 g/mL. A liquid chromatography–mass spectrometer (LCMS) was used to investigate the primary constituents of the *Polygonatum sibiricum* compound using an ACQUITY UPLC HSS T3 column (2.1 mm × 100 mm × 1.8 µm, Waters, Milford, MA, USA). The mobile phase consisted of an aqueous phase of 0.1% formic acid in water and an organic phase of acetonitrile, with a flow rate of 0.3 mL/min, a column temperature of 35 °C, and an injection volume of 0.5 μL. Mass spectrometry analysis was conducted using an electrospray ionization source (ESI) with a nozzle voltage of 4 kV in positive ion mode and 3.5 kV in negative ion mode. The sheath gas temperature was established at 350 °C, with a flow rate of 11 L/min; the drying gas flow rate was 5 L/min, and the drying gas temperature was set at 300 °C. The mass spectrometry scanning mode used a complete scan with a mass-to-charge ratio range of *m*/*z* 100 to 1700, a cone voltage of 100 V, and a collision energy gradient of 10, 20, 40, and 60 eV. The collected raw data were submitted to Qualitative Analysis 10.0 software, where the unknown chemical identification procedure was configured using its wizard settings and method templates, and the original data were subjected to peak extraction. The characteristic peaks in the sample were examined, and potential molecular formulas were derived by extracting the molecular ion chromatographic peaks and isotope peaks. Secondary fragments were compared with the PCDL secondary database and the *Polygonatum sibiricum* plant-derived component library, and results with a quality deviation exceeding 7.5 ppm and a matching score below 70 points. The secondary fragmentation results were compared with the online database SIRIUS version 5.8.5, leading to the selection of compounds with matching scores exceeding 80 and FingerID below 50. As shown in Figure 1, A total of 27 characteristic peaks from 28 samples of *Polygonatum sibiricum*, *Poria cocos*, *Lilium brownii*, and *Radix Glycyrrhizae Preparata* were identified. The analysis revealed the presence of active ingredients in *Polygonatum sibiricum*, including flavonoids and sugars, as well as the primary medicinal components of *Lilium brownii*, *Radix Glycyrrhizae Preparata*, and *Poria cocos*, such as various saponins and sugars. Compounds like jolsonin 7-O-β-D glucoside, jolsonin, king lily glycoside, and licorice saponin were successfully matched in the plant library of *Polygonatum sibiricum*, *Lilium brownii*, and *Radix Glycyrrhizae Preparata*, confirming that the medicinal solution contained the active ingredients of these plants.

#### 2.3.2. Animals and Experimental Groups

Following a 7-day regimen of consistent feeding, 96 mice were randomly allocated into six groups based on body weight: a blank control group (distilled water), a CRS model group (distilled water), a positive drug group (fluoxetine 10 mg/kg, fluoxetine was dissolved in distilled water to prepare a solution containing 1 mg of fluoxetine per mL.), and three PSF dosage groups (samples from Section 2.3.1 were taken and diluted with distilled water to the following three concentrations: 1.365 g/kg, 2.73 g/kg, and 5.46 g/kg), with a daily gavage volume of 10 mL/kg corresponding to the mice’s body weight. During the restraint period, mice in each group were fasted from food and water; they had free access to food and water at other times.

#### 2.3.3. Establishment of Chronic Restraint Model

**Figure 2:**
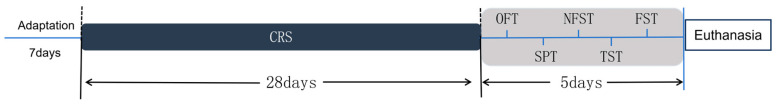
Flow chart of chronic restraint model establishment and behavioral detection experiment (OFT: open-field experiment; SPT: sugar water preference experiment; NSFT: novel inhibition of feeding experiment; TST: hanging tail experiment; FST: forced swimming experiment).

Mice were placed in a cylindrical restraint device (10 × 2.5 cm), which included a fixed base that permitted head movement but prevented complete rotation, while ensuring adequate air circulation. Continuous restraint was conducted for 28 days, with 10 h of confinement per day. Behavioral assessments (twelve animals were randomly selected from each group of sixteen animals) were performed after model establishment, and no restraint was applied during the assessments. During the 5 days of behavioral tests, drugs were administered daily before the behavioral tests. (The experimental flow chart is shown in Figure 2).

#### 2.3.4. Open-Field Test (OFT)

The open-field experiment, developed by Hall in 1934 [26], is often used to assess the autonomous motor skills and exploratory behavior of animals. Before performing trials, animals were transferred to the testing room one hour in advance. The open-field apparatus comprised a cylinder with a lower diameter of 28 cm, an upper diameter of 30 cm, and a height of 28 cm, with the cylinder’s color contrasting that of the animal (white rat with a black tube, black rat with a white tube). Each animal was placed in the middle of the cylinder for assessment, allowing it to move freely for 5 min. The image recognition and tracking method was used to record and analyze the movement distance and duration of animals in real time to evaluate the motor function of mice. The machine was sanitized with 75% alcohol after each test.

#### 2.3.5. Sucrose Preference Test (SPT)

In accordance with the methodologies of Spanagel and Schweizer et al. [27,28], as well as the aggregation of prior experiments conducted by this group, the experiments were divided into a sugar water adaptation phase and a testing phase. During this period, subjects were provided with one bottle of 1% sucrose solution and one bottle of distilled water, with the positions of the bottles altered every 12 h to mitigate positional bias. The testing was conducted two days after the adaptation phase. The unique inhibitory feeding experiment was conducted after a 24 h fasting period (without water) in mice.

#### 2.3.6. Novelty-Suppressed Feeding Test (NSFT)

The feeding-suspended experiment was assessed by Britton in 1981 [29]. Based on the aggregation of prior experiments conducted by this research group [30], the animals were moved to the experimental room one hour in advance. Similar-sized food was placed at the center of the test box (50 cm × 36 cm × 20 cm), and the mice were oriented with their backs to the box. The duration of the feeding incubation period was recorded over a span of five minutes, with a duration of five minutes noted if the mice did not consume any food within that timeframe.

#### 2.3.7. Tail Suspension Test (TST)

The TST is a well-established behavioral approach for assessing the efficacy of antidepressant medications, measuring their effects based on the duration of animal immobility [31]. Fix the tail of the mouse with already-prepared tape, ensuring to leave a loop for attaching the hook. The tape should be positioned approximately 1 cm from the tail’s tip, with its length adjustable as needed. The mouse should be suspended smoothly on the “S” shaped iron hook within the test box, with its head positioned 5–10 cm above the bottom of the test box, maintaining a vertical orientation. The sensor on the tail box bracket must be connected to the system, which will automatically record the duration of the mouse’s immobility. The overall duration of the experiment was 6 min, excluding the first 2 min adaptation phase, during which the cumulative immobility time of the mice was recorded and analyzed for the subsequent 4 min after suspension.

#### 2.3.8. Forced Swim Test (FST)

FST, first developed by Porsolt in 1978, is used as both an acute model and a behavioral assessment tool for evaluating animal despair. Place the experimental animal in a forced swimming apparatus maintained at a constant temperature of 24 ± 1 °C, with a water depth of 15 cm (the container measures 20 cm in height and 14 cm in diameter). The animal will initially swim vigorously in an attempt to escape; however, as the trial continues, it will become floating and motionless, ultimately falling to a state of “behavioral despair.” The test duration was 6 min, with the computer automatically recording the cumulative immobility time of the mice for the last 4 min after the first 2 min acclimation period.

#### 2.3.9. Sample Collection

Upon completion of the behavioral test, the hippocampal and cortical tissues had been extracted from the ice after denecking, transferred to cryopreservation tubes, and preserved in liquid nitrogen. Hippocampal and cortical tissue samples were preserved at −80 °C until retrieved for further analysis.

#### 2.3.10. UPLC-MS/MS Detection

According to this group’s earlier experimental methodologies [32], tissue neurotransmitter levels were measured using ultrafast high-performance liquid chromatography and a QTRAP 5000 mass spectrometer(AB Sciex, Framingham, MA, USA). A gradient wash was conducted on a RESTEK ultrawater C18 column(Restek Corporation, Bellefonte, PA, USA) to isolate metabolites.

#### 2.3.11. Fluorescence Quantitative PCR (qPCR) Experiments

Mouse hippocampus tissue was extracted from −80 °C, and 1 mL of TRIzon was added to every 30–50 mg of tissue after weighing. The mixture was thoroughly homogenized in an ice water bath, vortexed, and allowed to stand at room temperature for 5 min. Chloroform was added, vortexed well, and allowed to stand at room temperature for 2 to 3 min. The mixture was centrifuged at 12,000 RPM at 4 °C for 15 min, then the supernatant was gently aspirated into a 1.5 mL RNase-Free centrifuge tube. An equal amount of isopropyl alcohol was added, vortexed well, and allowed to stand at room temperature for 10 min. Then the mixture was centrifuged at 12,000 rpm at 4 °C for 10 min, the supernatant was removed, then the RNA was washed three times with 75% ethanol, dried, and 25 μL of RNase-Free ddH2O was added to dissolve the RNA. RNA concentrations were measured using the Nanodrop 2000(Thermo Fisher Scientific, Wilmington, DE, USA) and then standardized using RNase-Free ddH_2_O. Reagents were added as per the kit instructions to create a 20 μL system. The system was incubated at 42 °C for 15 min using a T100 thermal cycler, then inactivated at 85 °C for 5 s to produce reverse transcription cDNA. Upon completion of the reaction, the expression level of the target gene was assessed using the 2^−∆∆CT^ technique, with GAPDH serving as the housekeeping gene.

#### 2.3.12. Western Blot Experiment

After the homogenization of hippocampal tissue, it was incubated on ice for 30 min, then centrifuged at 12,000 RPM at 4 °C for 30 min to collect the supernatant. The protein concentration was assessed using the BCA method. An appropriate volume of the protein supernatant was aliquoted to prepare a protein solution at 5 μg/μL using protein lysate and SDS-PAGE Loading Buffer (5×), and the supernatant was subsequently stored at −80 °C after boiling. Proteins were isolated by the SDS-PAGE technique with a 10% polyacrylamide gel, and thereafter transferred to a nitrocellulose membrane (NC membrane) under a continuous current of 300 mA in an ice-water bath. The protein membrane was blocked at ambient temperature using 5% skim milk, subsequently removed, and washed three times with TBST. It was then incubated overnight at 4 °C with diluted primary antibodies (GSDMD, 1:1000; NLRP3, 1:1000; Caspase1, 1:1000; IL-18, 1:1000; IL-1β, 1:1000; β-actin, 1:4000) and further incubated for 1.5 h at room temperature with an HRP-labeled secondary antibody (1:5000). Following three washes with TBST, the secondary antibody was incubated for one hour at ambient temperature. Protein bands were seen using the BeyoECL PLUS/MOON Kit(Beyotime Biotechnology, Shanghai, China), and grayscale values of band density were assessed using Image J software(ImageJ 1.x).

#### 2.3.13. HE Staining

After cardiac perfusion, the murine cerebral tissue was removed and immersed in 4% paraformaldehyde for future use, followed by dehydration in a 30% sucrose solution. Subsequent to dehydration, the designated brain region was removed, mounted, pre-cooled in a cryotome for 30 min, and the brain tissue was maintained in a vertical position, affixed to the base. Mouse brain tissue was sectioned into coronal slices measuring 20 μm in thickness. The dissected brain slices were placed on a 24-well plate filled with cryopreservation solution and refrigerated at −20 °C for future use. The removed brain tissue slices were subjected to HE staining solution at room temperature for 5 min, rinsed quickly in distilled water, decolorized in 75% and 95% ethanol successively, and treated with anhydrous ethanol three times for 5 min each. The areas were washed with xylene twice for 5 min each. The section was sealed with neutral resin and allowed to cure in a fume chamber, after which the slide was examined under a light microscope, and the image was recorded.

#### 2.3.14. Nissl Staining

The removed brain tissue slices were subjected to Nissl staining solution at room temperature for 5 min, rinsed quickly in distilled water, decolorized in 75% and 95% ethanol successively, and treated with anhydrous ethanol three times for 5 min each. The areas were washed with xylene twice for 5 min each. The section was sealed with neutral resin and allowed to cure in a fume chamber, after which the slide was examined under a light microscope, and the image was recorded.

#### 2.3.15. Immunofluorescence Experiments

Brain tissue slices (20 μm thick) were washed with 0.01 M PBS three times for 5 min each. Bovine serum protein (5%) was mixed with 0.2% Triton X-100(Sigma-Aldrich, St. Louis, MO, USA) and blocked for 2 h at room temperature. Sealed sections were immersed in pre-diluted primary antibodies and incubated overnight on a low-temperature shaker. The primary antibody was recovered by washing the sections three times with 0.01 M PBS. The sections were immersed in the secondary antibody and incubated at room temperature for 2 h, away from light. The sections were washed three times with 0.01 M PBS protected from light. The brain slices were put on a slide, dried, and sealed with a fluorescence quenching solution for examination under a fluorescence microscope.

#### 2.3.16. Microbial Diversity Sequencing (16s-rRNA)

Mouse fecal samples were collected under sterile conditions, immediately deposited in sterile centrifuge tubes, and stored at −80 °C after quick-freezing with liquid nitrogen. After genomic DNA extraction, 1% agarose gel electrophoresis was used to identify the isolated genomic DNA, and the V3–V4 variable region of the 16S rRNA gene was amplified by PCR using 338F_806 primers. The amplification products were purified, recovered, and quantified using the QuantiFluor-ST™ Blue Fluorescence Quantitation System (Promega, Madison, WI, USA). High-throughput sequencing was carried out using Illumina’s PE250 platform(Illumina Inc., San Diego, CA, USA). Bioinformatics study of the sequencing data: Non-repeating sequences were grouped at 97% similarity to produce operational taxonomy (OTUs). OTU analysis was used to determine the microbiota composition and structural information of each sample group at various categorization levels. Samples were examined for alpha diversity using the Chao1 index, Shannon index, and other markers. Microbiota diversity and richness were compared among groups. The data were shown using non-metric multidimensional scale (NMDS) and principal coordinate analysis (PCoA), as well as β-diversity analysis to demonstrate structural changes in microbial communities among groups. The Wilcoxon rank-sum test was performed to compare the various species at the genus level.

### 2.4. Statistical Analysis

SPSS 26.0 was used for statistical analysis, while GraphPad Prism 9.5 was used for graphing. The experimental data were the mean ± standard error of measurement (mean ± SEM). When the data were normal, the one-way analysis of variance, the LSD technique for uniform variance, and the T3 test for uneven variance were employed. Nonparametric tests are used when there is a deviation from the normal state, and a *p*-value of <0.05 indicates statistical significance.

## 3. Results

### 3.1. Behavioral Results

**Figure 3:**
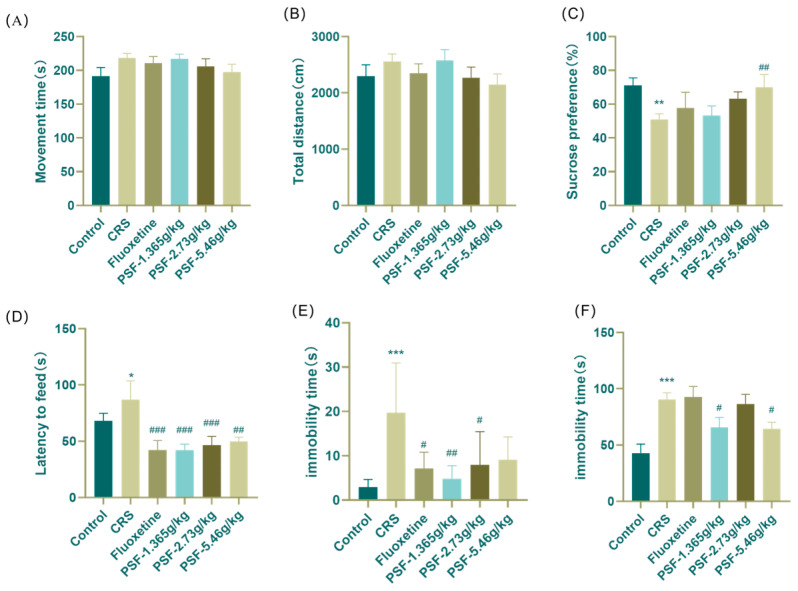
Effects of PSF on behavioral experiments of CRS mice. (**A**) Effects of PSF on the movement distance of CRS mice in open-field experiments; (**B**) effect of PSF on exercise time in open-field experiments in CRS mice; (**C**) effect of PSF on sugar water preference experiment in CRS mice; (**D**) effect of PSF on novel inhibitory feeding experiment in CRS mice; (**E**) effect of PSF on the suspension of the tail of CRS mice; (**F**) effect of PSF on forced swimming experiment in CRS mice (*n* = 8~11, Mean ± SEM, compared with blank control group, * *p* < 0.05, ** *p* < 0.01, *** *p* < 0.01; compared with the model group, ^#^ *p* < 0.05, ^##^*p* < 0.01, ^###^ *p* < 0.01).

After four weeks of restriction, Figure 3A,B show that no significant differences in movement distance or exercise duration were observed between the groups (F_(5,55)_ = 0.882, *p* > 0.05; F_(5,56)_ = 1.103, *p* > 0.05), indicating the absence of central excitation or inhibitory effects at the administered reagent dose, and that voluntary movement in mice remained unaffected. Figure 3C–F show that in comparison to the blank control group, the sugar water preference index in the model group was markedly diminished (*p* < 0.01), the feeding latency was significantly prolonged (F_(5,47)_ = 5.757, *p* < 0.05), and the immobility duration during tail suspension and forced swimming was dramatically augmented (*p* < 0.001; F_(5,47)_ = 6.238, *p* < 0.001). Compared with the model group, the sugar water preference index of the PSF (5.46 g/kg) group exhibited a significant increase (*p* < 0.01). The feeding latency period for the PSF (1.365 g/kg, 2.73 g/kg, 5.46 g/kg) group was markedly reduced (F_(5,47)_ = 5.757, *p* < 0.001, *p* < 0.001, *p* < 0.01). Additionally, the PSF (1.365 g/kg, 2.73 g/kg) group demonstrated a significant decrease in immobility time of TST (*p* < 0.01, *p* < 0.05). Furthermore, the forced swimming immobilization time for mice in the PSF (1.365 g/kg, 5.46 g/kg) group was significantly diminished (F_(5,47)_ = 6.238, *p* < 0.05, *p* < 0.05).

### 3.2. Effect of PSF on the Relative Expression of Inflammatory Factor mRNA in the Hippocampus of CRS Mice

**Figure 4:**
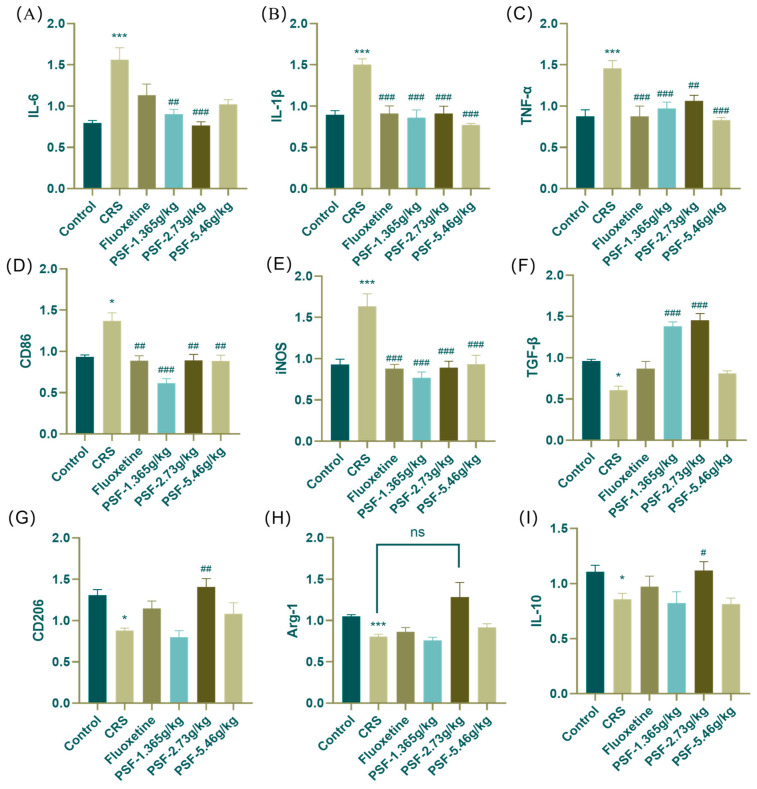
(**A**–**E**) Effect of PSF on the relative expression of pro-inflammatory factor mRNA in hippocampal tissue of CRS mice: (**A**) IL-6; (**B**) IL-1β; (**C**) TNF-α; (**D**) CD86; (**E**) iNOS. (**F**–**I**) Effect of PSF on the relative expression of anti-inflammatory factor mRNA in hippocampal tissue of CRS mice: (**F**) TGF-β; (**G**) CD206; (**H**) Arg-1; (**I**) IL-10; (*n* = 6~7, mean ± SEM, compared with the blank control group, * *p* < 0.05, *** *p* < 0.001; compared with the model group, ^#^ *p* < 0.05, ^##^ *p* < 0.01, ^###^ *p* < 0.001; ns: not significant).

Figure 4A–E shows that mice in the model group had significantly higher mRNA expression of pro-inflammatory markers IL-6, IL-1β, TNF-α, CD86, and iNOS in the hippocampus (*p* < 0.001; F_(5,33)_ = 14.367, *p* < 0.001; F_(5,34)_ = 8.329; *p* < 0.001; *p* < 0.05; F_(5,34)_ = 10.861, *p* < 0.001) than the blank control group. Treatment with PSF (1.365 g/kg) significantly reduced the expression of pro-inflammatory markers IL-6, IL-1β, TNF-α, CD86, and iNOS in the hippocampus of CRS mice compared to the model group (*p* < 0.01; F_(5,33)_ = 14.367, *p* < 0.001; F_(5,34)_ = 8.329, *p* < 0.001; *p* < 0.001; F_(5,34)_ = 10.861, *p* < 0.001). Treatment with PSF (2.73 g/kg) significantly reduced the mRNA expression of pro-inflammatory markers IL-6, IL-1β, TNF-α, CD86, and iNOS in the hippocampus of CRS mice (*p* < 0.001; F_(5,33)_ = 14.367, *p* < 0.001; F_(5,34)_ = 8.329, *p* < 0.01; *p* < 0.01; F_(5,34)_ = 10.861, *p* < 0.001). After PSF therapy (5.46 g/kg), the levels of pro-inflammatory proteins IL-1β, TNF-α, CD86, and iNOS mRNA in the hippocampus of CRS mice were much lower (F_(5,33)_ = 14.367, *p* < 0.001; F_(5,34)_ = 8.329, *p* < 0.001; *p* < 0.01; F_(5,34)_ = 10.861, *p* < 0.001). Figure 4F–I show that mice in the model group had significantly lower mRNA expression of anti-inflammatory markers TGF-β, CD206, Arg-1, and IL-10 in their hippocampus compared to the blank control group (*p* < 0.05; *p* < 0.05; F_(5,32)_ = 6.291, *p* < 0.001; F_(5,34)_ = 3.384, *p* < 0.05). After PSF (1.365 g/kg) dosage intervention, CRS mice showed significantly higher mRNA expression of anti-inflammatory factor TGF-β in their hippocampus compared to the model group (*p* < 0.001). After PSF (2.73 g/kg) dosage intervention, the CRS mice’s hippocampal relative mRNA expression of anti-inflammatory proteins TGF-β, CD206, and IL-10 increased significantly (*p* < 0.001; *p* < 0.01; F_(5,34)_ = 3.384, *p* < 0.05), and Arg-1 expression also increased.

### 3.3. Effect of PSF on the Expression of Pyroptosis-Related Factor (NLRP3/caspase1/GSDMD) Protein in Hippocampal Tissue of CRS Mice

**Figure 5:**
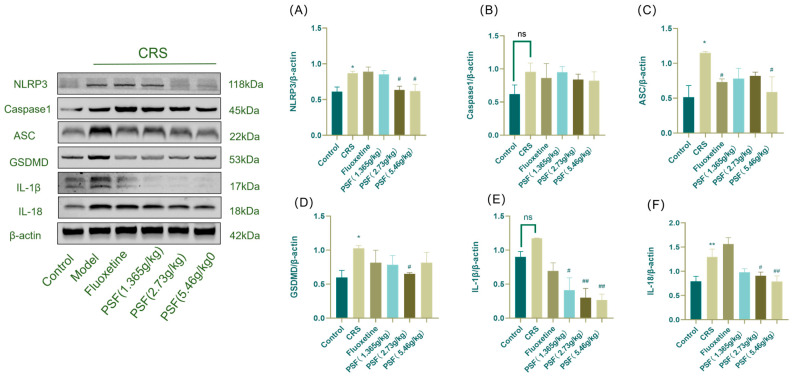
Effect of PSF on the expression of proteins related to the hippocampal NLRP3/Caspase1/GSDMD signaling pathway in CRS mice. (**A**) Western blot detection of hippocampal NLRP3/β-actin; (**B**) Casepase1/β-actin; (**C**) ASC/β-actin; (**D**) GSDMD/β-actin; (**E**) IL-1β/β-actin; (**F**) IL-18/β-actin content (n = 3, mean ± SEM, compared with the blank control group, * *p* < 0.05, ** *p* < 0.01; compared with the model group, ^#^ *p* < 0.05, ^##^ *p* < 0.01; ns: not significant).

As shown in Figure 5, the model group had significantly higher expression levels of NLRP3, ASC, GSDMD, and IL-18 in their hippocampus tissue compared to the blank control group (*p* < 0.01; F_(5,12)_ = 2.885, *p* < 0.05; F_(5,12)_ = 1.543, *p* < 0.05; F_(5,12)_ = 7.341, *p* < 0.01). In addition, Caspase1 and IL-1β levels increased. After PSF (1.365 g/kg) therapy, IL-1β expression significantly reduced in CRS mice compared to the model group (*p* < 0.05). PSF (2.73 g/kg) significantly reduced the expression levels of NLRP3, GSDMD, IL-1β, and IL-18 in CRS mice (*p* < 0.05; F_(5,12)_ = 1.543, *p* < 0.05; *p* < 0.01; F_(5,12)_ = 7.341, *p* < 0.05). PSF (5.46 g/kg) administration significantly reduced the expression levels of NLRP3, ASC, IL-1β, and IL-18 in the hippocampus of CRS mice (*p* < 0.05; F_(5,12)_ = 2.885, *p* < 0.05; *p* < 0.01; F_(5,12)_ = 7.341, *p* < 0.01).

### 3.4. Effect of PSF on Tryptophan Metabolism in Hippocampal Tissues of CRS Mice

**Figure 6:**
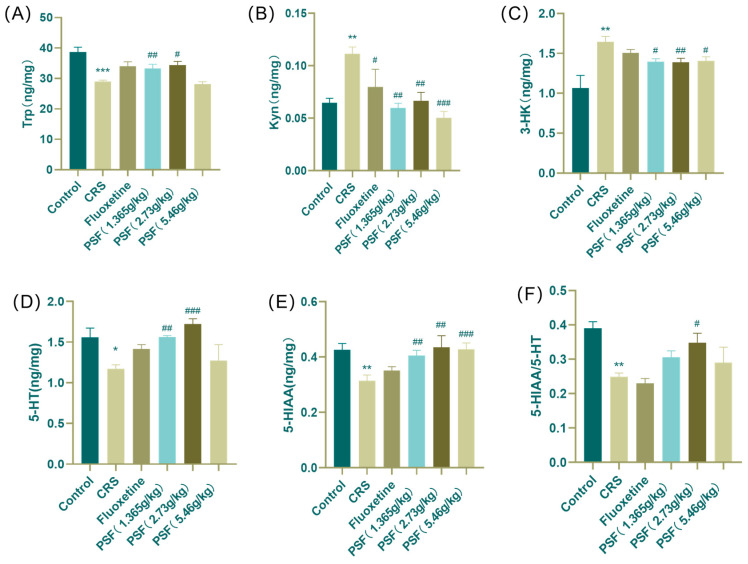
Effect of PSF on tryptophan metabolite content in the hippocampus of CRS mice. (**A**) Trp content in hippocampus; (**B**) Kyn content in the hippocampus; (**C**) 3-HK content in the hippocampus; (**D**) 5-HT content in the hippocampus; (**E**) 5-HIAA content in the hippocampus; (**F**) 5-HIAA/5-HT ratio (*n* = 8~10, mean ± SEM, compared with the blank control group, * *p* < 0.05, ** *p* < 0.01, *** *p* < 0.001; compared with the model group, ^#^ *p* < 0.05, ^##^ *p* < 0.01, ^###^ *p* < 0.001).

As shown in Figure 6, the model group of mice had considerably lower levels of Trp, 5-HT, 5-HIAA, and 5-HIAA/5-HT in their hippocampus compared to the blank control group (F_(5,43)_ = 9.894, *p* < 0.001; *p* < 0.05; *p* < 0.01; *p* < 0.01). Kyn and 3-HK levels increased dramatically (*p* < 0.01). After PSF (1.365 g/kg) treatment, CRS mice showed significantly higher levels of Trp, 5-HT, and 5-HIAA in their hippocampus (F_(5,43)_ = 9.894, *p* < 0.01; *p* < 0.01; *p* < 0.01), but Kyn and 3-HK levels dropped (*p* < 0.01; *p* < 0.05). Treatment with PSF (2.73 g/kg) resulted in significant increases in Trp, 5-HT, and 5-HIAA levels in the hippocampus of CRS mice (F_(5,43)_ = 9.894, *p* < 0.05; *p* < 0.001; *p* < 0.01; *p* < 0.05), while Kyn levels dropped. PSF (5.46 g/k) treatment enhanced 5-HIAA levels in CRS mice (*p* < 0.001) while decreasing Kyn and 3-HK levels (*p* < 0.001; *p* < 0.05).

### 3.5. Effects of PSF on Neurotransmitters in Hippocampal Tissues of CRS Mice

**Figure 7:**
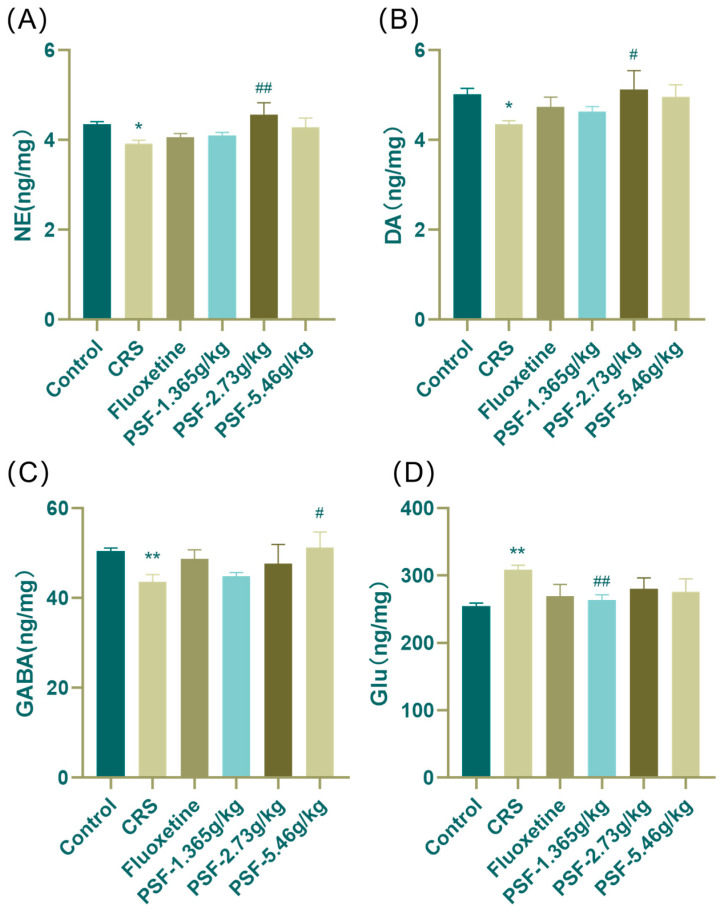
Effects of PSF on neurotransmitter content in the hippocampus and cortex of CRS mice. (**A**) NE content in hippocampus; (**B**) DA content in the hippocampus; (**C**) GABA content in the hippocampus; (**D**) Glu content in the hippocampus (*n* = 8~10, mean ± SEM, compared with the blank control group, * *p* < 0.05, ** *p* < 0.01; compared with the model group, ^#^ *p* < 0.05, ^##^ *p* < 0.01).

As shown in Figure 7, mice in the model group had considerably lower levels of NE, DA, and GABA in their hippocampus compared to the blank control group (F_(5,43)_ = 2.608, *p* < 0.05; F_(5,46)_ = 1.575, *p* < 0.05; *p* < 0.01), and Glu levels increased dramatically (F_(5,44)_ = 2.062, *p* < 0.01). After PSF (1.365 g/kg) dosage intervention, CRS mice had significantly lower Glu content in their hippocampus compared to the model group (F_(5,44)_ = 2.062, *p* < 0.01). PSF (2.73 g/kg) treatment significantly elevated NE and DA levels in the hippocampus of CRS mice (F_(5,43)_ = 2.608, *p* < 0.01; F_(5,46)_ = 1.575, *p* < 0.05). PSF (5.46 g/kg) dosage intervention significantly enhanced GABA levels in the hippocampus of CRS mice (*p* < 0.05).

### 3.6. Effect of PSF on Microglia (Iba-1) in CRS Mice

**Figure 8:**
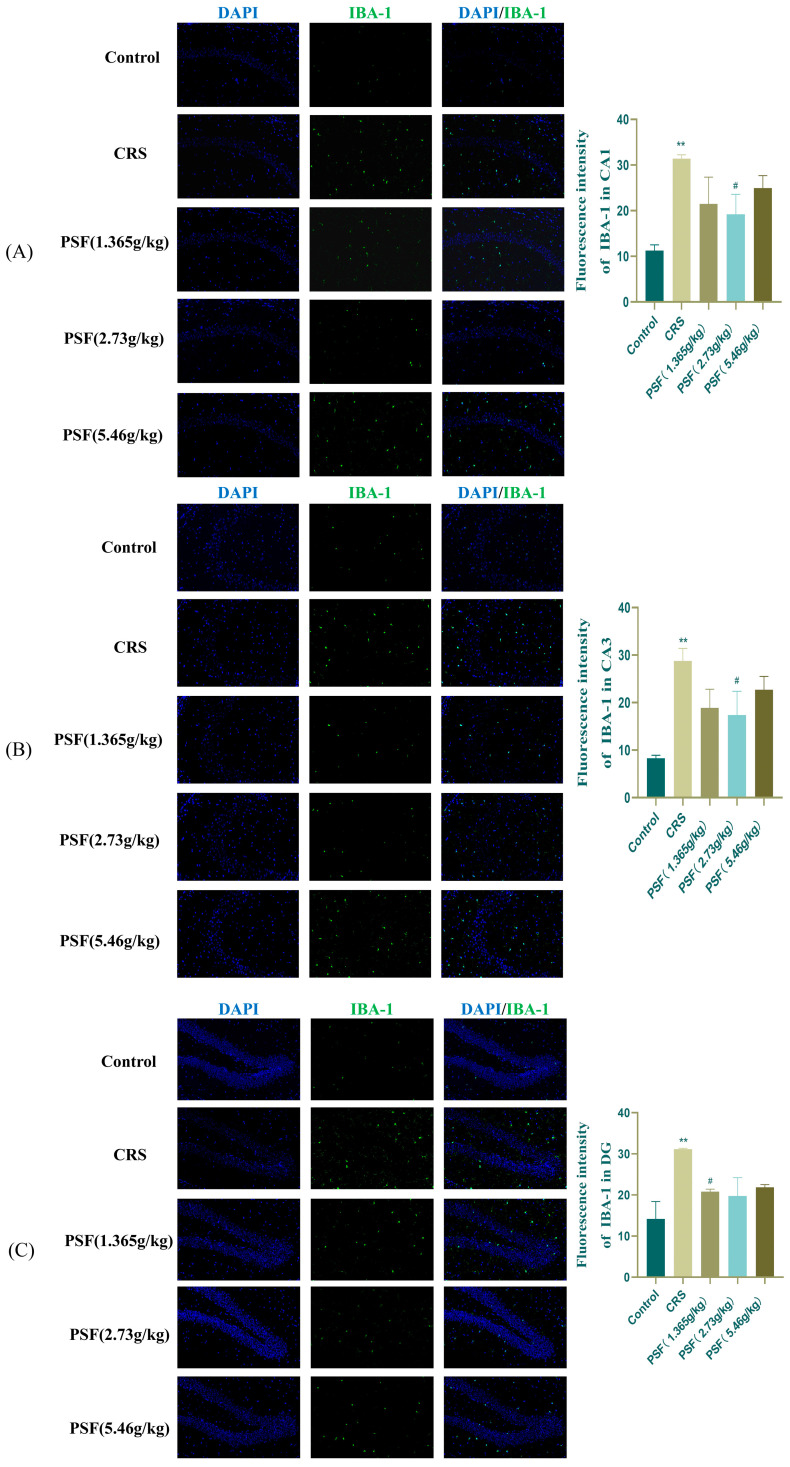
Regulatory effect of PSF on the microglial marker Iba-1 in CRS mice (20x). (**A**) Immunofluorescence results in the CA1 region and the number of Iba-1 positive cells; (**B**) immunofluorescence results in the CA3 region and the number of Iba-1 positive cells; (**C**) immunofluorescence results in the DG region and the number of Iba-1 positive cells. (n = 3, Mean ± SEM, compared to the blank control group, ** *p* < 0.01, compared to the model group, ^#^ *p* < 0.05).

As shown in Figure 8, immunofluorescence analysis revealed that CRS significantly increased Iba-1 expression in the hippocampal CA1, CA3, and DG regions of mice (F_(5,12)_ = 3.298, *p* < 0.01; F_(5,12)_ = 3.610, *p* < 0.01; *p* < 0.01), while PSF (1.365 g/kg) treatment significantly decreased Iba-1 expression in the hippocampal DG region (*p* < 0.01). PSF (2.73 g/kg) dosage intervention significantly reduced Iba-1 expression in the hippocampus CA1 and CA3 regions (F_(5,12)_ = 3.298, *p* < 0.05; F_(5,12)_ = 3.610, *p* < 0.05).

### 3.7. Effects of PSF on CRS Mouse Neurons

**Figure 9:**
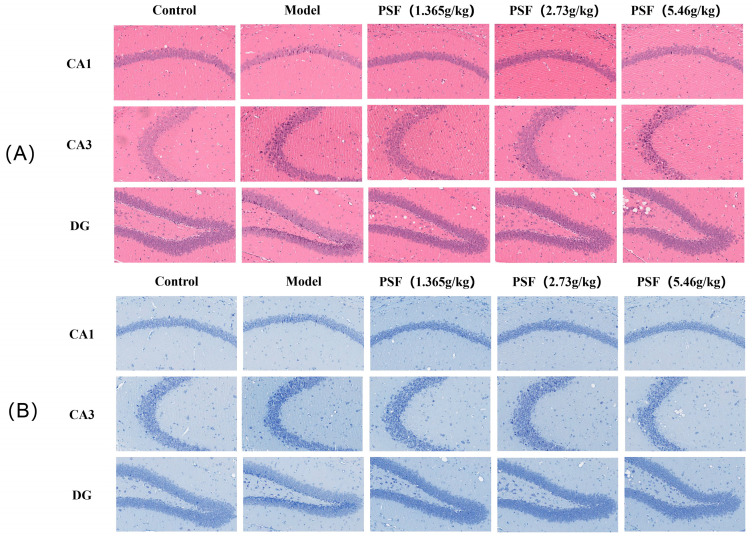
Effect of PSF on CRS mouse neurons (20×): (**A**) HE staining results; (**B**) Nissl staining results.

After HE staining, the cytoplasm exhibits a peach–red color, while the nucleus is colored blue–purple. Figure 9A illustrates that the cell volume of pyramidal neurons in the hippocampal CA1, CA3, and DG regions of mice within the CRS model group was diminished, exhibiting significant cell loss, irregular cellular arrangement, widespread neuronal loss, and episodes of necrosis, alongside pronounced extracellular voids, all of which were markedly abnormal compared to the blank control group. After PSF injection, the structure of neuronal cells in the CA1, CA3, and DG regions of the treatment group remained intact and organized with only a minimal incidence of neuronal cell nucleus degeneration or necrosis. Nissl can indirectly respond to the functional state of neurons. As illustrated in Figure 9B, the neurons in the CA1, CA3, and DG areas of the hippocampus are consistently and tightly organized, with Nistenstein bodies prominently observable. In comparison to blank controls, CRS resulted in neuronal injury, a disorganized structure of the hippocampal CA1, CA3, and DG areas in mice, and a decrease in neuron number, along with the disintegration of Nielsen bodies. The number of neurons in the hippocampus CA1, CA3, and DG areas of CRS animals was augmented, and the disorganized arrangement of neurons was ameliorated after PSF treatment.

### 3.8. Effect of PSF on the Gut Microbiota of CRS Mice

#### 3.8.1. OTU Cluster Analysis of Mouse Fecal Microbiota

**Figure 10:**
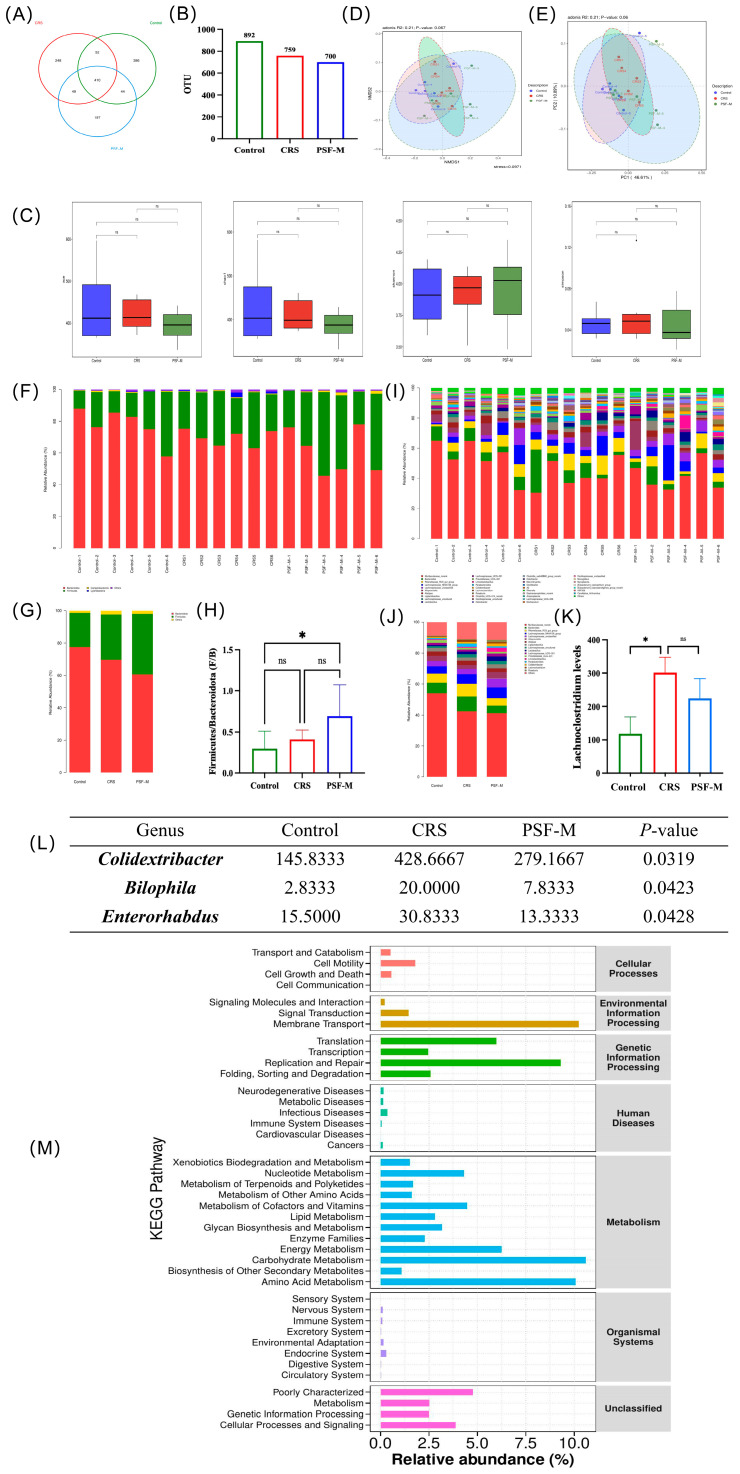
Effects of PSF on intestinal microbiota of CRS mice (n = 6, compared with the blank control group, * *p* < 0.05; ns: not significant) (**A**,**B**) Venn diagram of operational taxa (OTUs) in different treatment groups: (**A**) Venn diagram; (**B**) histogram of the number of OTUs in different treatment groups. (**C**–**E**) Alpha and Beta diversity analysis: (**C**) ACE, Chao 1, Shannon, and Simpson indices; (**D**) NMDS analysis; (**E**) PCoA analysis. (**F**–**H**) Histogram of species distribution at the level of fecal flora phylum of mice after different treatments: (**F**) histogram of species distribution of fecal flora in each mouse; (**G**) histogram of the distribution of fecal microbiota species in each treatment group; (**H**) fecal firmicutes/bacteroidetes ratios of mice in each treatment group. (**I**–**K**) Horizontal species distribution of fecal microbiota in mice after different treatments: (**I**) histogram of species distribution of fecal microbiota in mice; (**J**) histogram of the distribution of fecal microbiota species in each treatment group; (**K**) relative abundance of manure Alistipes in mice in each treatment group; (**L**) Wilcoxon rank sum test analysis of fecal microbiota of mice after different treatments; (**M**) KEGG functional prediction analysis (data analysis was conducted using the control and model groups).

The Venn diagram derived from OTU analysis illustrates the number of unique and overlapping OTUs among various sample groups, hence visualizing the sample composition at the OTU level. In the control group, CRS group, and PSF-M(PSF(2.73 g/kg)) group, there were 410 overlapping OTUs, with 248, 386, and 197 unique OTUs in the control group, CRS group, and PSF-M group, respectively (Figure 10A,B).

#### 3.8.2. Alpha and Beta Diversity Analysis of Fecal Microbiota in Mice

Both samples were examined for alpha and beta varieties. Figure 10C indicates that there are no substantial variations in the ACE index, Chao1 index, Shannon index, and Simpson index across the various treatment groups. To further assess differences among sample groups, the beta diversity of the microbiota was evaluated. Figure 10D illustrates that the NMDS1 comparison analysis produced R2 = 0.21 and *p* = 0.061, signifying no significant variation in microbiota composition among the treatment groups. Figure 10E illustrates that the comparative analysis of PC1 produced R2 = 0.21 and *p* = 0.06, indicating no significant variation in bacterial composition across the treatment groups.

#### 3.8.3. Composition of Fecal Microbiota in Mice

At the phylum level, the flora of each treatment group primarily was composed of Bacteroidota, Firmicutes, Campylobacterota, and Cyanobacteria (Figure 10F,G). In comparison to the control group, the Firmicutes/Bacteroidetes (F/B) ratio in the CRS group tended to rise, whereas the ratio in the PSF-M group showed a significant increase (*p* < 0.05) (Figure 10H). At the genus level, the flora of each treatment group mainly consisted of Muribaculaceae_norank, Bacteroides, Rikenellaceae_RC9_gut_group, Lachnospiraceae_NK4A136_group, and unclassified Lachnospiraceae (Figure 10H,I). In comparison to the control group, the relative abundance of Lachnoclostridium in the CRS group was considerably elevated (*p* < 0.05), whereas the relative abundance of Lachnoclostridium diminished after PSF-M therapy (Figure 10K).

#### 3.8.4. Analysis of Differential Microbiota in Mouse Feces

The differential microorganisms of the fecal microbiota of mice in each group are presented at the genus level as 10(L). In comparison to the control group, the relative abundance of Colidextribacter, Bilophila, and Enterorhabdus in the feces of mice in the CRS group was significantly elevated (*p* < 0.05), whereas the relative abundance of these three genera approached the levels observed in the control group after PSF-M treatment.

#### 3.8.5. KEGG Enrichment Pathway Analysis of Fecal Microbiota in Mice

Figure 10M illustrates that the functional categories associated with environmental information processing, genetic information processing, and metabolism are prominently represented, signifying that the mouse gut microbiome is in a highly active, rapidly proliferating, and adaptive state. These microbiotas play a crucial role in the host’s nutrient digestion, energy acquisition, and metabolite production.

## 4. Discussion

Chronic stress is an important risk factor for depression and is recognized for inducing maladaptive neuroimmune activation and metabolic remodeling in the brain. The current work demonstrated that CRS induced characteristic depressive-like symptoms, such as anhedonia and behavioral despair, alongside neuroinflammation, microglial hyperactivation, neuronal damage, and disruptions in tryptophan metabolism. We illustrate that PSF significantly mitigates these aberrations and produces strong antidepressant-like effects.

Chronic restraint stress (CRS) is a well-established experimental model that predominantly provokes anhedonia and behavioral depression. The current study demonstrated that chronic restraint stress (CRS) significantly reduced sucrose preference and heightened immobility in both the tail suspension and forced swim tests, alongside an extended latency in the novelty-suppressed feeding test, signifying the manifestation of fundamental depressive-like characteristics, such as anhedonia, behavioral despair, and stress-induced motivational inhibition. However, no significant differences in locomotor distance or movement duration were detected in the open-field test, indicating that neither CRS nor PSF substantially influenced locomotor activity. These data demonstrate that PSF effectively alleviates chronic stress-induced depression-like phenotypes.

Chronic stress is a significant environmental element that triggers depression, mostly by enhancing the inflammatory response of the central nervous system, notably through the overactivation of microglia and the secretion of inflammatory mediators [33]. Prolonged chronic stress activates microglia. Activated microglia enhance the expression of the microglial marker Iba-1, resulting in peripheral nerve tissue damage and neuronal death [34,35]. This study reported a significant reduction in Iba-1 expression in the hippocampal CA1, CA3, and DG areas after PSF intervention. Concurrently, the mRNA expression of pro-inflammatory factors IL-6, IL-1β, TNF-α, iNOS, and the M1 microglial marker CD86 in the hippocampus of mice within the CRS model group exhibited a significant rise, whereas the expression of anti-inflammatory factors, including TGF-β, CD206, Arg-1, and IL-10, diminished. After PSF administration, these inflammatory markers were markedly ameliorated. This indicates that PSF may mitigate neuroinflammation and subsequent neuronal injury by suppressing microglial hyperactivation and diminishing the secretion of pro-inflammatory mediators.

Prolonged microglial activation during chronic stress can exacerbate neuroinflammation via inflammasome-mediated pyroptotic signaling. Pyroptosis is an inflammatory form of programmed cell death mediated by Gasdermin protein, whose activation relies on the assembly of NLRP3 inflammasomes and the activation of caspase-1, resulting in the maturation and release of pro-inflammatory molecules such as IL-1β and IL-18. NLRP3 inflammasomes are among the largest and most prominent inflammasomes, comprising NLRP3, apoptosis-associated speck-like protein (ASC), and caspase-1. This investigation revealed that CRS markedly increased the protein expression of NLRP3, caspase-1, ASC, GSDMD, IL-1β, and IL-18 in the hippocampus, while PSF successfully inhibited the expression of these proteins. Consequently, PSF may mitigate neuroinflammation by inhibiting NLRP3-mediated pyroptosis and diminishing the secretion of pro-inflammatory molecules.

The dysregulation of the tryptophan–kynurenine pathway indicates a significant metabolic outcome of persistent neuroinflammation. Tryptophan (Trp) metabolism serves as a crucial hub linking the immunological, neurological, and metabolic systems [36]. Tryptophan (Trp) is predominantly metabolized via two pathways: approximately 5% is transformed into serotonin (5-HT), while the majority is transported into the kynurenine (Kyn) route via indoleamine 2,3-dioxygenase (IDO) or tryptophan 2,3-dioxygenase (TDO) [37]. Chronic stress and inflammation can activate IDO/TDO, shifting tryptophan metabolism towards the kynurenine pathway, resulting in diminished serotonin synthesis and the production of neurotoxic metabolites such as 3-hydroxykynurenine (3-HK) [12], which subsequently cause neuronal damage and depressive behavior [38].

This study indicated that the levels of Trp, 5-HT, 5-HIAA, and the ratio of 5-HIAA/5-HT in the hippocampus of CRS mice were dramatically diminished, whilst the concentrations of Kyn and 3-HK were elevated, suggesting an association with enhanced kynurenine pathway metabolism. After PSF intervention, both Trp and 5-HT levels increased, whereas Kyn and 3-HK levels decreased. These data demonstrate that PSF is associated with restored tryptophan metabolism under stress conditions, which may be related to reduced inflammation-related IDO/TDO activity, thereby linking its anti-inflammatory effects to subsequent neurochemical and behavioral improvements.

Disruption of tryptophan metabolism and neuroinflammatory signaling ultimately leads to neurotransmitter imbalance and neuronal dysfunction. Decreased serotonin availability immediately disrupts monoaminergic neurotransmission, an essential pathological characteristic of depression, while elevated kynurenine metabolites further intensify excitatory–inhibitory dysregulation by enhancing glutamatergic hyperactivity and diminishing GABAergic tone [39,40,41]. This study demonstrated that the concentrations of norepinephrine (NE), dopamine (DA), serotonin (5-HT), and its metabolite 5-hydroxyindoleacetic acid (5-HIAA) in the hippocampus of mice in the chronic restraint stress (CRS) model group were markedly diminished, but the levels of these neurotransmitters were partially restored following pharmacological stress-free (PSF) intervention. PSF also regulates the state of balance of glutamic acid (Glu) and γ-aminobutyric acid (GABA). The excitatory neurotransmitter Glu and the inhibitory neurotransmitter GABA work together to regulate emotions and behavior [42]. Abnormally elevated glucose levels can induce excitatory neurotoxicity, subsequently contributing to depression [43]. Individuals with depression frequently exhibit hyperactivity of glutamate and inadequate functioning of the GABAergic system, resulting in neuronal excitotoxicity and network disruption [44]. CRS animals exhibited elevated hippocampus glutamate levels and reduced GABA, while PSF intervention restored this pattern. Recovery of GABA/Glu imbalance promotes in reestablishing neuronal homeostasis and may correlate with enhancements in behavioral hopelessness and depression-like symptoms. The current study demonstrated that CRS-induced activation of the kynurenine pathway resulted in diminished hippocampal concentrations of serotonin (5-HT), norepinephrine (NE), and dopamine (DA), alongside elevated glutamate (Glu) and decreased gamma-aminobutyric acid (GABA). After PSF treatment, levels of 5-HT, NE, and DA increased, while Glu decreased and GABA increased. Significantly, these neurochemical alterations were accompanied by the structural preservation of hippocampal neurons, as demonstrated by HE and Nissl staining. Chronic restraint stress results in disruption and a diminished quantity of neurons in the hippocampal CA1, CA3, and DG areas, indicating considerable neuronal damage. Following PSF injection, there were more Nissl bodies and a more regular neuronal organization, suggesting that it had neuroprotective effects.

Ultimately, we investigated whether PSF also affected gut microbiota, which is now considered a significant regulator of neuroimmune and metabolic homeostasis [45,46]. Chronic stress can disturb the homeostasis of intestinal flora, elevate intestinal permeability, and facilitate the penetration of inflammatory substances into the bloodstream, thereby impacting the functionality of the blood–brain barrier and neuroinflammation [38]. The results obtained from 16S rRNA sequencing in this study indicated that while CRS did not markedly alter the α and β diversity of the microbiota, it induced specific modifications at the genus level, including an increase in the relative abundance of Lachnoclostridium, which subsequently decreased after PSF intervention. The prevalence of Colidextribacter, Bilophila, and Enterorhabdus in the CRS group was markedly elevated, and these genera exhibited a trend of recovery after PSF intervention. Lachnoclostridium and Bilophila have been linked to intestinal inflammation and metabolic disorders in previous studies [47,48]. Bilophila is a harmful bacterium in the intestine, and its increase can elevate the risk of gastrointestinal inflammatory responses [49]. Enterorhabdus is a genus of potential anti-inflammatory and neuroprotective bacteria [50]. However, Enterorhabdus in the gut microbiota of mice in the CRS group exhibited an abnormal increase, which may be a compensatory response of the gut microecosystem to CRS-induced intestinal inflammation and barrier damage, where the gut attempts to maintain microecological stability by increasing the abundance of anti-inflammatory genera. After PSF intervention, as intestinal inflammation subsides and microecological balance is restored, the abundance of Enterorhabdus shows a recovery trend, which further confirms the regulatory effect of PSF on the gut microecosystem. The specific mechanism of action of the genus Colidextribacter in intestinal inflammation and depression has not yet been fully elucidated. Nevertheless, its significant enrichment in the CRS group and the recovery trend after PSF intervention suggest that it may be involved in the pathological processes of CRS-induced gut microecological imbalance and depression, and its specific role still requires further verification through subsequent mechanistic experiments. Notably, PSF intervention can effectively reverse the CRS-induced abnormal changes in these genera, regulate the structure of gut microbiota, and restore the balance of the gut microecological environment. The regulating influence of PSF on intestinal flora may be linked to its abundant polysaccharides, saponins, and other constituents, which can act as prebiotics to enhance the proliferation of beneficial bacteria or suppress the growth of pathogenic bacteria [51]. KEGG function prediction study indicated that the microbiota exhibited increased activity in glucose and amino acid metabolism after PSF intervention, implying its potential influence on host health through the regulation of microbiota metabolic functions. Although these alterations were minor, they may enhance the systemic anti-inflammatory and metabolic features of PSF, hence indirectly boosting its neuroprotective and anti-depressive effects.

This study still has certain drawbacks. First, the experiment only used male ICR mice; no female animals were included, making it impossible to assess the impact of sex differences on PSF efficacy. Secondly, the components that are essential in PSF and the interaction mechanisms among these components require additional analysis. In future studies, we will carry out quantitative verification, reference standard research, reproducibility assessment, and identification of defined marker compounds. Third, the direct link between alterations in gut microbiota and brain function requires validation through fecal transplantation, metabolomics, and further methodologies. Fourth, the current clinical human dose (*Polygonatum sibiricum* (9 g), *Poria cocos* (15 g), *Lilium brownii bulb* (15 g), and roasted *Glycyrrhiza uralensis* (3 g)) translates to 5.46 g/kg in mice, while 2.46 g/kg produced more comprehensive antidepressant effects. Further optimization of the human-equivalent dose is needed in future studies. In addition, the sample size for some protein analyses was relatively small (n = 3), and the LSD post hoc test was used for multiple comparisons. These limitations should be considered when interpreting the results.

## 5. Conclusions

The present study demonstrates that the Polygonatum-based formula (PSF) successfully mitigates chronic restraint stress-induced depressive-like behaviors through the coordinated regulation of neuroimmune and metabolic pathways. PSF mitigates microglial hyperactivation and NLRP3-mediated pyroptosis, thereby reducing the production of neuroinflammatory cytokines and preventing subsequent dysregulation of tryptophan–kynurenine metabolism and neurotransmitter homeostasis. Collectively, these findings provide a molecular rationale for the development of PSF as a functional food-derived strategy for the prevention and adjunctive management of stress-related depression by maintaining hippocampus neuronal integrity and restoring neurochemical balance.

## Figures and Tables

**Table 1:** Gene sequences.

Gene	Primers
GAPDH	Forward: AACAGCAACTCCCACTCTTC
	Reverse: CCTGTTGCTGTAGCCGTATT
IL-6	Forward: TTCTTGGGACTGATGCTGGTGAC
	Reverse: GTGGTATCCTCTGTGAAGTCTCCTC
IL-1β	Forward: CTCGCAGCAGCACATCAACAAG
	Reverse: CCACGGGAAAGACACAGGTAGC
TNF-α	Forward: TGTCTACTCCCAGGTTCTCTT
	Reverse: GCAGAGAGGAGGTTGACTTTC
CD86	Forward: TATCTGCCGTGCCCATTTAC
	Reverse: GTGCTCGTACAGAACCAACT
iNOS	Forward: GGAATCTTGGAGCGAGTTGT
	Reverse: CCTCTTGTCTTTGACCCAGTAG
TGF-β	Forward: GCTTCTCCCAAGTGTGTCAT
	Reverse: GACTGCTGGTGGTGTATTCTT
CD206	Forward: GGCGAGCATCAAGAGTAAAGA
	Reverse: CATAGGTCAGTCCCAACCAAA
Arg-1	Forward: TCATGGAAGTGAACCCAACTC
	Reverse: CGAAGCAAGCCAAGGTTAAAG
IL-10	Forward: TTGAATTCCCTGGGTGAGAAG
	Reverse: TCCACTGCCTTGCTCTTATTT

## Data Availability

The original contributions presented in this study are included in the article. Further inquiries can be directed to the corresponding authors.

## Abbreviations

The following abbreviations are used in this manuscript:

Abbreviations	Full Name
PSF	*Polygonatum sibiricum*-based functional formula
PSF-M	PSF (2.73 g/kg)
CRS	Chronic restraint stress
SPT	Sucrose preference test
NSFT	Novelty-Suppressed Feeding Test
TST	Tail Suspension Test
FST	Forced Swim Test
NE	Norepinephrine
DA	Dopamine
GABA	γ-aminobutyric acid
Glu	Glutamic acid
Trp	Tryptophan
Kyn	Kynurenine
3-HK	3-hydroxy-L-kynurenine
5-HT	5-hydroxytryptamine
5-HIAA	5-hydroxyindole-3-acetic acid
IL-6	Interleukin-6
IL-1β	Interleukin-1beta
TNF-α	Tumor necrosis factor-alpha
CD86	Cluster of differentiation 86
iNOS	Inducible nitric oxide synthase
TGF-β	Transforming growth factor-beta
CD206	Cluster of differentiation 206
Arg-1	Arginase-1
NLRP3	NOD-like receptor family, pyrin domain-containing 3
Caspase1	Cysteinyl aspartate-specific protease 1
ASC	Apoptosis-associated speck-like protein containing a CARD
GSDMD	Gasdermin D
IL-18	Interleukin-18
Iba-1	Ionized calcium-binding adapter molecule 1
